# Fibrosis: from mechanisms to novel treatments

**DOI:** 10.1186/s41232-023-00314-1

**Published:** 2024-01-02

**Authors:** Akihiko Yoshimura

**Affiliations:** Department of Microbiology and Immunology, Keio University School of Medicine, 35 Shinanomachi, Shinjyuku-Ku, Tokyo, 160-8582 Japan

Fibrosis is a pathological hallmark in the majority of chronic inflammatory ailments, characterized by the excessive accumulation of extracellular matrix (ECM) components, including collagen and fibronectin. The etiology of fibrosis is diverse, encompassing hereditary inflammatory disorders, persistent infections, repeated exposure to toxins, irritants, and smoke, autoimmune inflammation, and rejection in transplantation. A shared characteristic in these fibrotic diseases is the activation of ECM-producing myofibroblasts, a central player in the remodeling of fibrotic tissue. Collagen deposition is a crucial and typically reversible facet of wound healing, severe or recurrent tissue injuries, or dysregulation in wound healing, it is necessary for the progression of normal tissue repair into an irreversible fibrotic state.

Gastrointestinal fibrosis is characterized as a state of heightened biological entropy resulting from aberrant tissue repair reactions. Acute or chronic inflammation in the gastrointestinal tract, notably in conditions such as inflammatory bowel disease, particularly Crohn’s disease, triggers fibrosis and stricture, often necessitating surgical or endoscopic intervention. Myocardial infarction, elevated serum cholesterol, obesity, poorly managed diabetes, and hypertension also contribute to fibrosis in various cases, with chronic inflammation playing a pivotal role in most instances.

This special edition centers on gastrointestinal fibrosis (Dr. Mikami’s article) and cardiac fibrosis (Dr. Ieda’s article), aiming to disseminate information on recent developments in fibrosis and its therapeutic approaches.

Dr. Mikami delves into the contemporary understanding of the pathogenesis of gastrointestinal fibrosis, concentrating on Crohn’s disease and fibrosis arising from stenosis post-endoscopic submucosal dissection (ESD). The article addresses significant challenges in developing antifibrotic agents and highlights key fibrotic pathways that have progressed to clinical trials [1].

The incidence of heart disease in Japan is on the rise due to lifestyle westernization and an aging population. Regenerative medicine emerges as a promising treatment option when conventional therapies are inadequate. However, stem cell limitations have prompted the exploration of “direct myocardial initialization” as an alternative treatment. Myocardial regeneration involves the in situ trans-differentiation of cardiac fibroblasts into cardiomyocytes. Dr. Ieda and collaborators have identified that three cardiomyogenic transcription factors, Gata4, Mef2c, and Tbx5 (GMT), can directly reprogram fibroblasts into induced cardiomyocytes (iCM) in mice. In humans, additional factors such as Mesp1 and Myocd are required. The article outlines factors that inhibit or promote reprogramming, with ongoing improvements for human applications, paving the way for further advancements in cardiac initialization research [2].
